# Risk factor analysis, antimicrobial resistance and pathotyping of *Escherichia coli* associated with pre- and post-weaning piglet diarrhoea in organised farms, India

**DOI:** 10.1017/S0950268819000591

**Published:** 2019-04-11

**Authors:** O. R. VinodhKumar, B. R. Singh, D. K. Sinha, B. S. Pruthvishree, Shika Tamta, Z. B. Dubal, R. Karthikeyan, Ramkumar N. Rupner, Y. S. Malik

**Affiliations:** 1 Division of Epidemiology, ICAR – Indian Veterinary Research Institute, Bareilly, India; 2 Division of Veterinary Public Health, ICAR – Indian Veterinary Research Institute, Bareilly, India; 3 Division of Biological Standardization, ICAR – Indian Veterinary Research Institute, Bareilly, India

**Keywords:** Antibiotic resistance, *E. coli*, pathotype, piglet diarrhoea, risk factors

## Abstract

A cross-sectional study was conducted from 2014 to 2017 in 13 organised pig farms located in eight states of India (Northern, North-Eastern and Southern regions) to identify the risk factors, pathotype and antimicrobial resistance of *Escherichia coli* associated with pre- and post-weaning piglet diarrhoea. The data collected through questionnaire survey were used to identify the risk factors by univariable analysis, in which weaning status, season, altitude, ventilation in the shed, use of heater/cooler for temperature control in the sheds, feed type, water source, and use of disinfectant, were the potential risk factors. In logistic regression model, weaning and source of water were the significant risk factors. The piglet diarrhoea prevalence was almost similar across the regions. Of the 909 faecal samples collected (North – 310, North-East – 194 and South – 405) for isolation of *E. coli*, pathotyping and antibiotic screening, 531 *E. coli* were isolated in MacConkey agar added with cefotaxime, where 345 isolates were extended spectrum *β*-lactamase (ESBL) producers and were positive for *bla*CTX-M-1 (*n* = 147), *bla* TEM (*n* = 151), *qnr*A (*n* = 98), *qnr*B (*n* = 116), *qnr*S (*n* = 53), *tet*A (*n* = 46), *tet*B (*n* = 48) and *sul*1 (*n* = 54) genes. Multiple antibiotic resistance (MAR) index revealed that 14 (2.64%) isolates had MAR index of 1. On the virulence screening of *E. coli*, 174 isolates harboured alone or combination of *Stx*1, *Stx*2, *eae*A, *hly*A genes. The isolates from diarrhoeic and post-weaning samples harboured higher number of virulence genes than non-diarrhoeic and pre-weaning. Alleviating the risk factors might reduce the piglet diarrhoea cases. The presence of multidrug-resistant and ESBL-producing pathogenic *E. coli* in piglets appears a public health concern.

## Introduction

Pig rearing plays a vital role in alleviating poverty and development of socio-economic condition in rural farming community in the developing Asian countries including India. The pig population of India is around 10.29 million as per 19th Livestock census, which constitutes about 2% of the ivestock population of India [1]. The development of a modern swine industry in India is indeed a need in recent years to negotiate the ever-increasing demand of animal protein but still majority of the pig rearers in the country do not have the sufficient knowledge about the pig production and their diseases. Piglet diarrhoea in neonatal and weaned piglets due to *Escherichia coli* is an economically important disease, affecting pigs during the first 2 weeks and post-weaning and is characterised by sudden death or diarrhoea, dehydration and growth retardation in surviving piglets [2, 3, 4].

Weaning is one of the important causes for piglet diarrhoea, which causes psychological, nutritional, environmental and physiological stress on piglets [5]. The other risk factors associated with piglet diarrhoea are pathogenic *E. coli*, stress, management factors and excessive feed intake [6, 7].

The enterotoxigenic *E. coli* (ETEC) colonises on intestinal epithelium by F4, F5, F6, F18 and F41 fimbriae attaching to specific receptors on the villous enterocytes and results in diarrhoea, dehydration, growth retardation and sometimes sudden death in piglets [3, 8–10].

In order to reduce the incidence of piglet diarrhoea, piglets are often treated with antibiotics. The indiscriminate and non-judicial use of antibiotics in piggery is also one of the causes for the emergence of resistant *E. coli* [11, 12]. Extended-spectrum *β*-lactamases (ESBLs) are a cluster of enzymes that exist in *Enterobacteriaceae* family members, especially in *E. coli* that facilitates the resistance to most *β*-lactams approved in human and veterinary medicine. In the recent times, the quick emergence and spreading of ESBL-positive *E. coli* isolates in food animals have been reported and gained huge attention globally due to their possible transmission through the food chain [13]. However, there are only limited reports stating the prevalence, pathotyping, virulence factors and risk factors for the occurrence of *E. coli* in neonatal and weaned piglets in India.

In this study, we assessed the risk factors of piglet diarrhoea, antimicrobial resistance pattern, pathotypes of *E. coli* associated with pre- and post-weaning diarrhoea in piglets from organised farms in India.

## Materials and methods

### Sampling design

**Fig. 1.**
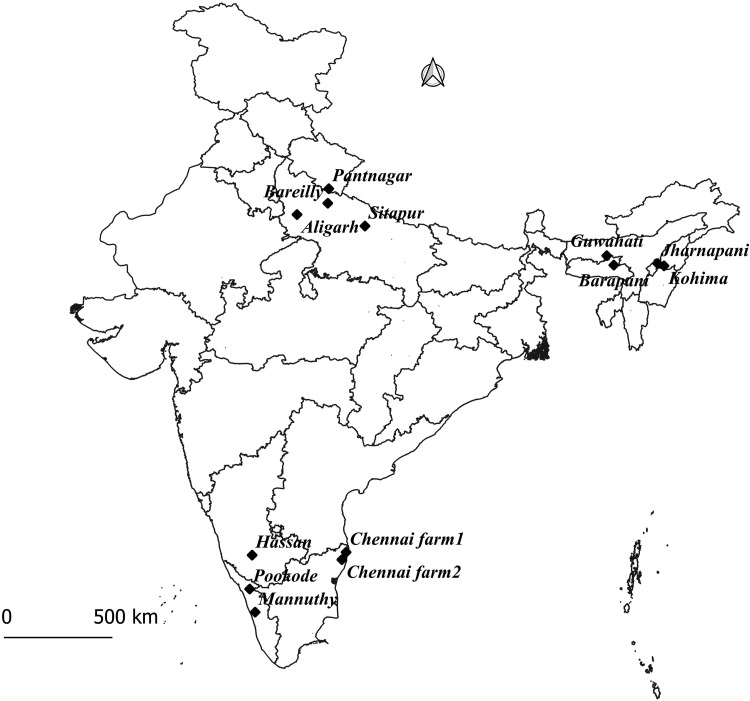
Sample collection locations for piglet diarrhoea (*N* = 13).

From August 2014 to July 2017, a total of 909 faecal samples were aseptically collected from 13 organised swine farms located in three regions (Northern, North-Eastern and Southern) covering eight states namely Assam, Meghalaya, Nagaland (North-Eastern states), Uttar Pradesh and Uttarakhand (Northern states) and Karnataka, Tamil Nadu and Kerala (Southern states) of India (Fig. 1). The selected states represent the major pig rearing pockets of North, North-East and Southern India [1]. The North-Eastern states have hilly terrain and subtropical climate, whereas Southern states have tropical climate. The Northern states lie mainly in the north temperate zone of the Earth, with cold winters, hot summers and moderate monsoons. A semi-structured peer-evaluated questionnaire (Supplementary file) was used for the collection of information about the demography of swine farm and husbandry practices, etc. The details of farm and number of samples collected were shown in Table 1. For each farm, the sample size calculations were carried out using Epitools software (http://epitools.ausvet.com.au/content.php?page=home) with 10–20% prevalence of piglet diarrhoea based on our preliminary study, 95% confidence interval and 80% power. Simple random sampling procedure with random number table was used in each farm to collect the faecal samples from pre- and post-weaning piglets, with and without diarrhoea, and were not treated with any antibiotics at least 2 weeks preceding the date of sample collection. A diarrhoeic case was considered when the piglet voided watery faecal material more than thrice a day, for at least 1 day. The diarrhoea was categorised based on frequency of defecation (3–5, >5 times/day), consistency of faeces (soft, watery, bloody, with or without mucus), and status of dehydration (severe, moderate, mild). The point prevalence of diarrhoea for each farm was calculated as the total number of piglets with diarrhoea at the time of sampling (numerator) divided by the total number of piglets available for sampling during that particular time (denominator). The faecal samples were collected aseptically using sterile swabs (HiMedia, India) and transported to the laboratory under cold chain. The questionnaire data are summarised in Supplementary Table S1.

**Table 1.** Details of the samples collected from different farms

Region	Location	Longitude	Latitude	No. of piglets available for sampling		No. of samples collected for isolation of *E. coli*	Sex		Weaning		Health status	
				Pre-wean	Post-wean		M	F	Pre-wean	Post-wean	Non-diarrhoeic	Diarrhoeic
Southern India (*n* = 405)	Hassan	76.1279	13.0210	60	45	70	41	29	58	12	55	15
	Mannuthy	76.2589	10.5306	210	95	171	80	91	119	52	152	19
	Pookode	76.0202	11.5412	121	45	88	42	46	71	17	78	10
	Chennai farm1	80.2310	13.1478	92	31	53	31	22	42	11	42	11
	Chennai farm2	80.04528	12.8231	58	18	23	14	9	18	5	19	4
Northern India (*n* = 310)	Bareilly	79.4320	28.3930	130	76	188	99	89	141	47	167	21
	Pantnagar	79.4760	29.0270	25	16	10	4	6	8	2	8	2
	Sitapur	81.0550	27.3970	128	45	71	33	38	52	19	61	10
	Aligarh	78.0880	27.8974	95	26	41	22	19	30	11	32	9
North Eastern India (*n* = 194)	Guwahati	91.6131	26.1017	95	35	53	24	29	43	10	43	10
	Jharnapani	93.8388	25.7585	70	42	48	26	22	27	21	44	4
	Barapani	91.9223	25.6906	115	36	62	30	32	46	16	48	14
	Kohima	94.1139	25.6585	40	24	31	15	16	22	9	25	6
	Total			1239	534	909	461	448	677	232	774	135

M, male; F, female

### Isolation and phenotypic characterisation of E. coli

The samples were suspended in 10 ml buffered peptone water and incubated for 6 h at 37 °C for pre-enrichment. Subsequent to enrichment in MacConkey broth for overnight at 37 °C, it was streaked on MacConkey agar added with cefotaxime (1 mg/l) and incubated at 37 °C for 18–24 h. From each plate, four lactose-fermenting colonies were picked up and streaked on eosin methylene blue agar (EMB) medium and incubated at 37 °C overnight for preliminary characterisation, and the isolates with metallic sheen were biochemically characterised.

### Antimicrobial susceptibility assay of E. coli isolates

The reference strains (Accession No: KT853018, KT867018, KT867020 and KT867021) were collected from the repository maintained at Division of Epidemiology, ICAR-Indian Veterinary Research Institute, Izatnagar to serve as control positive strains. The isolates were tested for antibiotic susceptibility pattern with amoxicillin (AMX, 10 µg), aztreonam (ATM, 30 µg), chloramphenicol (C, 30 µg), ceftriaxone (CRO, 30 µg), cefpodoxime (CPD, 10 µg), ceftazidime (CAZ, 30 µg), ceftazidime + clavulanic acid (CAZ-CLA, 30/ 10μg), cefotaxime (CTX, 30 µg), cefepime (FEP, 30 µg), cefixime (CFM, 5 µg), cefoxitin (FOX, 30 µg), cefotaxime + clavulanic acid (CTX-CLA, 30/10 µg), cefoperazone (CFP, 75 µg), tetracycline (TE, 30 µg), nitrofurantoin (F/M, 300 µg), gentamicin (GM, 10 µg), cotrimoxazole (COT, 25 µg), ciprofloxacin (CIP, 5 µg) and norfloxacin (NOR,10 µg) by using disk diffusion test [14]. The Clinical and Laboratory Standards Institute (CLSI), 2014 breakpoints were used for the interpretation of susceptibility pattern [15]. The *E. coli* isolates were screened by combination disk method for phenotypic confirmation of ESBL production [14]. Multidrug-resistant (MDR) strains (i.e. strains showing resistance to at least two groups of antibiotics) were identified. Multiple antibiotic resistance (MAR) index was calculated using the formula as total number of antibiotics to which the organism was resistant divided by the total number of antibiotics to which the organism was tested [16].

### PCR targeting antimicrobial resistance and virulence genes of E. coli

Genomic DNA was extracted from *E. coli* isolates by QIAamp DNA Mini Kit (Qiagen, Hilden, Germany) and PCR was performed for β-lactamase [17], sulphonamide resistance [18], plasmid-mediated quinolone resistance determinants [19], tetracycline resistance genes [20] and virulence markers for Shiga toxin [21]. The PCR was carried out in 25 µl reaction volume containing 2 µl of DNA template, 10 pmol/μl of each primer (1 µl), 2x DreamTaq PCR master mix (Thermo Fisher Scientific Baltics UAB, Lithuania, 12.5 µl) and nuclease-free water to make the volume of 25 µl. The PCR primers and cycle conditions were given in Supplementary Table S2. The amplified PCR products were resolved by electrophoresis on 1.5% agarose gel containing ethidium bromide (0.5 µg/l) (Molecular Bio Grade; Merck, Mumbai, India) with 100 bp ladder (Thermo Fisher Scientific). The gels were run at 100 V for 1.5 h in 1X TBE buffer and documented by the gel documentation system (UVP, UK).

### Statistical analysis

Information from the questionnaire were digitised into a Microsoft excel spreadsheet (Microsoft Corporation) and piglet diarrhoea results were coded as negative = 0 and positive = 1. The *χ*^2^ test or Fisher's exact test with Yates correction was used to test the associations between the predictor variables and the outcome variable. Fisher's exact test with Yates correction was used when expected cell frequencies were <5. In piglet diarrhoea model, the analysis of multiple predictors of pre- and post-wean diarrhoea was performed by multivariable logistic regression analysis using stepwise forward method considering only the factors with *P* ⩽ 0.2 in univariable analysis. In the final multivariable logistic regression model, only the factors significant at *P* ⩽ 0.05 level for Wald test were retained. The model fit was assessed by Hosmer and Lemeshow (HL) test. A mixed-effect model was created once the single-level model had been finalised in order to assess any impact of the region as a random effect [22].

## Results

This study reveals the point prevalence of *E. coli*-associated piglet diarrhoea on 13 pig farms from different regions of India along with the risk factor analysis, pathotyping and antimicrobial resistance in pre- and post-weaning piglets. The information collected through questionnaire revealed that all farms were negative for gastrointestinal helminths and coccidian oocysts. Farms practised routine screening for gastrointestinal helminths and regular deworming. The farms were classified based on the information collected and presented in Supplementary Table S1. Based on the area or size of the landholding, the farms were classified as small, medium and large (<100 acres – small; 100–300 acres – medium; >300 acres – large). Based on the number of pigs reared, the farms were classified as small (<200), medium (200–500) and large (>500). Majority of the farms reared pure and cross breeds of Landrace, Large White Yorkshire and Duroc. Few farms also reared native and cross breeds. Except one pig farm (Guwahati, Assam), other farms reared farm animals such as cattle, sheep and goat. Many of the farms provided heaters or coolers for temperature control. Seven farms used commercial feed and six farms used own mill ground feed. All farms used β-lactam and cephalosporin antimicrobials for treating sick animals. In general, all the farms had cement floor with regular disinfectant cleaning and ventilated animal shed. In common, weaning was practiced between 35 and 45 days. No outbreak of any contagious disease was recorded over the last 12 months. There was no dedicated handler to take care of diseased and healthy animals in all the farms.

The point prevalence of piglet diarrhoea ranged from 3.57% to 14.29%, was lowest (3.57%) at pig farm from Jharnapani, Nagaland (North-East) whereas highest (14.29%) from Hassan, Karnataka (South). There was no significant difference in point prevalence of piglet diarrhoea (*P* = 0.46) across the three regions.

The data analysis of 13 farms showed that the risk factors for diarrhoea were weaning status, season, altitude, ventilation, use of heater/cooler for temperature control in the sheds, feed type, water source, and use of disinfectant, (Table 2). The crude, strata-specific and adjusted odds ratio revealed that there was no confounding effect of sex and weaning status, while effect modification was noticed for sex. The post-weaning piglets were 3.7 times more prone to diarrhoea than pre-wean. Compared with monsoon, in winter piglets had 2.8 times higher risk of diarrhoea. The piglets reared in plain or low altitude had 1.8 times more risk for diarrhoea than piglets in hilly or high altitude. Use of shallow well water, commercial feed, poor ventilation and absence of temperature control mechanism were positively associated with piglet diarrhoea, while regular use of disinfectants reduced the piglet diarrhoeal cases. Logistic regression analysis of the factors having P ≤ 0.2 showed a predictive model with weaning and water source as significant risk factors (HL Test: χ^2^ 9.4; df = 8, P = 0.31; −2 Log likelihood = 660.703, Nagelkerke R^2^ = 0.183) (Table 3). The inclusion of region as a random effect in the final model resulted in a minor (<10%) alteration to the coefficients associated with each of the variables retained within the model and all variables remained statistically significant.

**Table 2.** Univariable analysis of statistically significant risk factors associated with piglet diarrhoea

Variables		Diarrhoeic	Non-diarrhoeic	*P* value	OR (95% CI)
Weaning	Post-wean	67	67	0.00**	3.73(2.54–5.44)
	Pre-wean	164	611		1 (Ref)
Season	Winter	51	147	0.00**	2.80 (1.83–4.26)
	Summer	27	177		1.23 (0.75–2.0)
	Monsoon	56	451		1 (Ref)
Altitude	Plain	254	521	0.00**	1.85 (1.19–2.86)
	Hilly	28	106		1 (Ref)
Ventilation	Fair	45	167	0.00**	1.84(1.24–2.74)
	Good	89	608		1 (Ref)
Water source	Shallow well	22	49	0.00**	3.64 (2.0–6.6)
	Spring	2	86		0.19(0.045–0.79)
	Bore and shallow well	67	291		1.87(1.24–2.83)
	Bore well	43	349		1 (Ref)
Feed	Commercial	473	302	0.00**	1.93 (1.34–2.79)
	Own mill	60	74		1 (Ref)
Presence of heater/cooler	No	76	312	0.00**	1.95 (1.34–2.82)
	Yes	58	463		1 (Ref)
Disinfectant	Weekly	56	242	0.00**	2.30 (1.5–3.52)
	Occasional	35	106		3.28 (2.0–5.38)
	Daily	43	427		1 (Ref)

Ref, reference category.

**P ⩽ 0.01.

**Table 3.** Risk factors associated with piglet diarrhoea in multivariable logistic regression model

Factors		Coefficient (*B*)	s.e.	*P* value	Exp (*B*)/odds ratio	Lower 95% CI	Upper 95% CI
Weaning	Post-wean	0	–	–	1 (Ref)	–	–
	Pre-wean	−1.517	0.222	0.00**	0.219	0.142	0.339
Water source	Spring	0	–	–	1 (Ref)	–	–
	Bore well	2.294	1.357	0.09	9.915	0.694	141.68
	Shallow well	4.418	1.451	0.00**	82.89	4.824	1424.54
	Both bore and shallow well	3.301	1.398	0.01**	27.135	1.750	420.625
Constant		−3.157	1.066	0.00**	–	–	–

Ref, reference category.

(Hosmer and Lemeshow Test: *χ*^2^ 9.4; df = 8; *P* = 0.31; −2 Log likelihood = 660.70, Nagelkerke *R*^2^ = 0.183).

**P ⩽ 0.01.

In North-Eastern region, the only risk factor associated with piglet diarrhoea was weaning, while the Southern and Northern regions showed weaning, presence of other animals, altitude of the farm, use of disinfectant, ventilation, water source, presence of heater or cooler, type of feed and season as risk factors (Supplementary Table S3).

On bacterial isolation, 531 ESBL-*E. coli* were isolated from 909 samples on cefotaxime-added MacConkey plates. The isolation rate of *E. coli* was less since they were selected against cefotaxime. Of the 774 non-diarrhoeic and 135 diarrhoeic faecal samples, 438 and 93 *E. coli*, respectively, were isolated. The isolation rate of *E. coli* from diarrhoeic samples (17.51%) was significantly higher than non-diarrhoeic samples (11.11%, *P* ⩽ 0.01). The *E. coli* (*n* = 531) were resistant to AMX (92.1%), CTX (82.11%), CAZ (80.41%), COT (31.83%), C (35.78%), GM (80.98%), TE (32.96%), F/M (30.32%), NX (64.97%), AZT (36.16%), CIP (66.67%), FEP (58.95%), CFM (55.37%), CRO (58.0%) and CEF (64.78%). With regard to MDR profiles of *E. coli* isolates (*n* = 531), 82.86% (*n* = 440) were resistant to three or more classes of antimicrobial agents. The *E. coli* from diarrhoeic samples showed significantly higher resistance to CTX, CAZ, C, COT, F/M and FEP than non-diarrhoeic samples (Supplementary Table S4). MAR index revealed 73 isolates (13.75%) with MAR index >0.2, while 14 (2.64%) isolates had MAR of 1 (i.e. resistant to all the antimicrobials tested). The MAR indices of the *E. coli* are given in Supplementary Table S5. The region-wise analysis showed that there was no significant difference in resistance pattern of *E. coli* isolates. The diarrhoeic samples harboured significantly higher number of drug-resistant isolates than non-diarrhoeic faecal samples. Based on combined disk method, 345 isolates were ESBL producers. These ESBL-producing isolates (*n* = 345) were positive for *bla*CTX-M-1 (*n* = 147), *bla* TEM (*n* = 151), *qnr*A (*n* = 98), *qnr*B (*n* = 116), *qnr*S (*n* = 53), *tet*A (*n* = 46), *tet*B (*n* = 48) and *sul*1 (*n* = 54) genes.

Virulence gene screening of the 438 *E. coli* of non-diarrhoeic and 93 *E. coli* of diarrhoeic samples revealed that 95 (21.7%) and 79 (84.9%) isolates, respectively harboured virulence genes. Similarly, out of 316 pre-wean and post-wean samples *E. coli*, 78 and 96 isolates harboured virulence genes, respectively. Of the *E. coli* from non-diarrhoeic piglets (*n* = 438), 51 (11.64%), 45 (10.27%), 43 (9.82%) and 49 (11.19%) harboured *Stx*1, *Stx*2, *eae*A and *hly*A genes, respectively. The *E. coli* isolates from diarrhoeic piglets (*n* = 93) harboured 23 (24.73%), 22 (23.66%), 16 (17.20%) and 20 (21.50%) of *Stx*1, *Stx2*, *eae*A and *hly*A genes, respectively. The *E. coli* from diarrhoeic samples harboured significantly higher number of virulence genes than non-diarrhoeic samples. Similarly, the post-wean samples harboured significantly higher number of virulence genes than pre-wean (Table 4). However, there was no significant difference in the distribution of virulence genes across the regions.

**Table 4.** Association of virulence factors with health and weaning status of piglets

Piglet status		No. of *E. coli* with virulence genes	*P* value	OR (95%CI)	No. of *E. coli* with *Stx*1	*P* value	OR (95%CI)	No. of *E. coli* with *Stx*2	*P* value	OR (95%CI)	No. of *E. coli* with *eae*A	*P* value	OR (95%CI)	No. of *E. coli* with *hly*A	*P* value	OR (95%CI)
Health status	Diarrhoeic	79	0.000***	20.4 (11.0–7.5)	23	0.001***	2.29 (1.43–4.34)	22	0.001***	2.70 (1.53–4.78)	16	0.04*	1.90 (1.02–3.56)	20	0.00**	2.18 (1.22–3.87)
	Non-diarrhoeic	95		1 (Ref)	51		1 (Ref)	45		1 (Ref)	43		1 (Ref)	49		1 (Ref)
Weaning	Post-wean	96	0.000***	2.46 (1.7–3.6)	31	0.01**	2..62 (1.24–5.5)	29	0.01**	2.64 (1.22–5.7)	28	0.03*	2.27 (1.01–4.8)	30	0.04*	2.08(1.01–4.28)
	Pre-wean	78		1 (Ref)	12		1 (Ref)	11		1 (Ref)	12		1 (Ref)	14		1 (Ref)

Ref, reference category.

*P ⩽ 0.05; **P ⩽ 0.01; ***P ⩽ 0.001.

## Discussion

Pig rearing plays a vital role in improving the livelihood of poor and marginal farmers of India. Production with minimum inputs and maximum output is the basis and requirement of the poor farmers. However, piglet diarrhoea is of great economic challenge to intensive pig farming and cause substantial economic losses [23]. Pre- and post-weaning piglet diarrhoea is a multi-factorial disease primarily attributed to *E*. *coli* [5, 23, 24]. It is commonly associated with the proliferation of *β*-haemolytic strains of ETEC in the small intestine [3] and frequently occurs within 2 weeks after weaning due to implications between the piglet, sow, environment and farm practices [25]. It also results into substantial economic losses in many swine herds due to 20–30% mortality in weaned piglets during acute outbreaks [2].

In the present study, point prevalence of piglet diarrhoea varied from 3.57% to 14.29%, across the locations surveyed. The region-wise prevalence of piglet diarrhoea was almost similar across the regions which indicates that piglet diarrhoea is one of the commonest problems throughout India. The occurrence of diarrhoea in post-weaned piglets was significantly higher than pre-weaned piglets. It may be due to the weaning stress, change in the physiological status and nutrition of the piglets during this period [5, 6]. The observations were in corroboration with Australian pig farms finding published recently [25]. Reports also state that this might be associated with the weaning stress, dietary changes and lack of antibodies due to withdrawal of sow's milk, which makes the piglets susceptible to commensal *E. coli* [3, 26]. The rate of isolation of *E. coli* from post-weaned diarrhoeic faecal samples was significantly higher than pre-weaned diarrhoeic faecal samples; the findings are in corroboration with Dutta *et al*. [27] from North-Eastern region. The higher rate of isolation of *E. coli* in post-weaning piglets might be due to stress, decrease in maternal antibody and lack of self-immunity [28]. In this study, a higher prevalence of *E. coli* in diarrhoeic piglets was observed (68.87%, 93/135) than non-diarrhoeic piglets (56.88%, 438/774). In piglets, diarrhoea is mainly associated with *E. coli* [4, 10] in pre- and post-weaning stages [29].

In the present study, the risk factors associated with piglet diarrhoea were weaning, season, ventilation, altitude, water source, feed, presence of heater/cooler and use of disinfectants. poor ventilation, harsh climatic conditions, absence of temperature control devices in the piglet sheds cause stress and may predispose the piglets to diarrhoea. The pig farms using shallow well water had more diarrhoea cases. Since shallow well has more chances for faecal contamination compared with deep bore wells [30]. Van Breda *et al*. [31] reported that bedding, temperature control in piglet pen and recent disease events were the risk factors associated with piglet diarrhoea on Australian pig farms. Weaning is a stressful phase in piglets, after weaning feed intake get reduced initially and the piglets may develop anorexia of variable duration and the extent varies between farms, depending on livestock management and the nature of the feed [32]. Hence investigating management practices to minimise the risk factors of pathogenic *E*. *coli* may help to cost reduction in the veterinary and medical care.

In the study, the occurrence of 64% (345/531) of ESBL-producing *E. coli* isolates might be associated with the selection of *E. coli* in cefotaxime-added media. The common use of *β*-lactam and cephalosporin antibiotics on the farms investigated may also contribute for ESBL-producing *E. coli*. In another study, the ESBL-producing *E. coli* was detected in 34 (56.7%) of 60 pigs, and 20.0% (eight of 40) of the pig farm worker's rectal swabs in China [33]. Our observations for isolation of higher proportion of ESBL-positive *E. coli* among piglets might be due to the fact that in earlier studies, selective *β*-lactam antibiotic(s) were not used in the isolation procedures. From India, ESBL-producing *E. coli* were reported in healthy piglets under organised and backyard piggery [34]. The carbapenem-resistant *E. coli* were reported in piglets of India [11]. Mandakini *et al*. [35] reported ESBL-producing Shiga toxigenic *E. coli* in piglet diarrhoea. The *E. coli* isolated from diarrhoeic piglets were comparatively more ESBL-positive than non-diarrhoeic piglets. Our results were in harmonious with the findings of Xu *et al*. [4], they reported high occurrence of ESBLs in sick animals. In the present study, virulent *E. coli* had lesser resistance for co-trimoxazole, nitrofurantoin, tetracycline and chloramphenicol compared with other antibiotics. However, earlier studies reported higher level of resistance to gentamicin, neomycin and sulphametoxazol-trimethoprim among virulent isolates of *E. coli* from diarrhoeic and non-diarrhoeic piglets [36]. This discrepancy might be associated with the overall decline in the use of these antibiotics in India since 2000 [37]. The antibiotic resistance pattern and MAR indices of our study were in concurrence with the earlier findings [4, 38]. Akwar *et al.* [39] reported MDR *E. coli* in weaner and finisher pigs.

In this study, out of the 531 *E. coli*, 174 isolates harboured any one of the virulence genes screened and the *E. coli* isolates from diarrhoeic piglets harboured significantly higher number of virulence genes in *E. coli* isolates than non-diarrhoeic piglets. The post-wean piglets harboured significantly higher number of virulence genes positive for *E. coli* compared with pre-wean piglets which was in corroboration with Van Breda *et al*. [31]. The distribution of virulence genes did not show any significant difference across the regions, this may be due to the ubiquitous nature of the *E. coli* in the environment. Pruthvishree *et al*. [11] reported carbapenem-resistant isolates harbouring *Stx*1, *Stx*2, *eae*A and *hly*A virulence genes. Furthermore, a significant statistical association between antimicrobial resistance and presence of virulence genes (*P* ⩽ 0.05) was seen. Association of antimicrobial resistance and virulence genes of *E. coli* from swine in Ontario, Canada has been reported previously [40]. Toledo *et al*. [41] hypothesised that the pathogenic *E. coli* presence in intestinal tract of healthy piglets may cause the disease due to the consequence of immune response induced by stress, temperature changes and diet. Besides, continuous shedding of pathogenic *E*. c*oli* into the environment through faeces might be responsible for the maintenance of a stable bacterial population, which contributes to the re-occurrence of disease in herds as well as potential public health threat due to possible transfer of ESBL organism to humans [42].

Even though this study describes the potential risk factors associated with piglet diarrhoea across India, it has certain limitations such as difference in agro climatic region, local management practices, feed ingredients used for feeding and viral agents associated with diarrhoea which were not taken in to consideration in this study.

## Conclusion

Piglet diarrhoea is one of the major causes of economic loss in pig farming. Tackling the risk factors associated with piglet diarrhoea may help in reducing the incidence. High ESBL-positive *E. coli* in faecal samples of diarrhoeic piglets with virulence genes warrants the establishment of antibiotics resistance surveillance programmes along with intensive research to develop alternatives to antimicrobials to ensure the high-level food safety standards to improve human and animal health.

## Supplementary material

For supplementary material accompanying this paper visit https://doi.org/10.1017S0950268819000591. click here to view supplementary material
