# Supplementary material for: Risk factor analysis, antimicrobial resistance and pathotyping of Escherichia coli associated with pre- and post-weaning piglet diarrhoea in organised farms, India
Source: Epidemiol Infect. 2019 Apr 11;147:e174. doi: 10.1017/S0950268819000591 (PMC6518820; doi:10.1017/S0950268819000591)
Supplement: Supplementary file 1 [file S0950268819000591sup001.docx]

**Supplementary table 1: Classification of questionnaire data based on farm characteristics (N=13)**

| **Characters** | | **No. of farms** |
| --- | --- | --- |
| Altitude | Plain | 9 |
|  | Hilly | 4 |
| Ventilation | Good | 9 |
|  | Fair | 4 |
| Use of Heater/cooler | Yes | 8 |
|  | No | 5 |
| Feed | Commercial | 7 |
|  | Own mill | 6 |
| Presence of other animals | Yes | 9 |
|  | No | 4 |
| Sheds in different heights | Yes | 2 |
|  | No | 11 |
| Use of beta-lactam antibiotics | yes | 4 |
|  | When needed | 9 |
| Use of cephalosporin antibiotics | Yes | 5 |
|  | No | 8 |
| Use of disinfectant | Daily | 4 |
|  | Weekly | 7 |
|  | When needed/occasional | 2 |
| Water source | Deep well | 6 |
|  | Shallow well | 1 |
|  | Both | 5 |
|  | Spring | 1 |
| Farm area (acre) | <100 | 3 |
|  | 100 to 400 | 4 |
|  | >400 | 6 |
| Farm size ( total number of animal holding) | <200 | 3 |
|  | 200 to 500 | 3 |
|  | >500 | 7 |

**Supplementary table 2: The primers and PCR cycling conditions used to amplify the antibiotic resistance and virulence genes of *E. coli* with expected amplicon size**

| **S. No** | **Gene** | **Primers sequence**  **(5’ to 3’)** | **Cyclical conditions** | **Product size (bp)** | **Reference** |
| --- | --- | --- | --- | --- | --- |
| 1 | *bla*CTXM | F- CAATGTGCAGCACCAAGTAA  R- CGCGATATCGTTGGTGGTG | 95°C X 5 m/95°C X 30 s – 65°C X 30 s – 72°C X 30s (5Cycles) 95°C X 30 s – 62°C X 30 s – 72°C X 30 s (10 Cycles)/95°C X 30s – 60°C X 30 s – 72°C X 30 s (15 Cycles)/95°C X 30 s – 58°C X 30 s – 72°C X 30s (15 Cycles)/72°C X 7 m | 540 | Dutta *et al*. (2013) |
| 2 | *Stx*1 | F- ATAAATCGCCATTCGTTGACTAC  R- AGAACGCCCACTGAGATCATC | 95°C x 5 m/95°C x45 s – 59°C x45 s – 72°C x1m (30 Cycles)/72°C x6 m | 180 | Paton and Paton (1998) |
| 3 | *Stx*2 | F- GGCACTGTCTGAAACTGCTCC  R- TCGCCAGTTATCTGACATTCTG | 95°C x 5 m/95°C x45 s – 59°C x45 s – 72°C x1m (30 Cycles)/72°C x6 m | 255 | Paton and Paton (1998) |
| 4 | *Sul1* | F- CGGCGTGGGCTACCTGAACG  R- GCCGATCGCGTGAAGTTCCG | 95°C X 5 m/ 95°C X 30 s – 65°C X 1 m – 72°C X 1 m (35 Cycles)/72°C X 7 m | 433 | Kerrn *et al*. (2002) |
| 5 | *qnrA* | F-ATT TCTCACGCCAGGATTTG  R-GATCGGCAAAGGTTAGGTCA | 95°C X 5 m/95°C X 30 s – 56°C X 1 m – 72°C X 1 m (35 Cycles)/ 72°C X 10 m | 516 | Ciesielczuk *et al*. (2013) |
| 6 | *qnrB* | F-GATCGTGAAAGCCAGAAAGG  R-ATGAGCAACGATGCCTGGTA | 95°C X 5 m/95°C X 30 s – 56°C X 1 m – 72°C X 1 m (35 Cycles)/ 72°C X 10 m | 476 | Ciesielczuk *et al*. (2013) |
| 7 | *qnrS* | F-GCAAGTTCATTGAACAGGGT  R-TCTAAACCGTCGAGTTCGGCG | 95°C X 5 m/95°C X 30 s – 56°C X 1 m – 72°C X 1 m (35 Cycles)/ 72°C X 10 m | 428 | Ciesielczuk *et al*. (2013) |
| 8 | *tetA* | F- GTGAAACCCAACATACCCC  R- GAAGGCAAGCAGGATGTAG | 95°C X 7 m/95°C X 1 m – 60°C X 1 m – 72°C X 1 m (35 Cycles)/ 72°C X 10 m | 888 | Maynard *et al.* (2004) |
| 9 | *tetB* | F- CCTTATCATGCCAGTCTTGC  R- ACTGCCGTTTTTTCGCC | 95°C X 7 m/95°C X 1 m – 60°C X 1 m – 72°C X 1 m (35 Cycles)/ 72°C X 10 m | 774 | Maynard et al. (2004) |

**Supplementary table 3: Region-wise significant risk factors associated with piglet diarrhoea**

| **Region** | **Variable** |  | **Diarrhoeic** | **Non-diarrhoeic** | **P value** | **OR ( 95 % CI)** |
| --- | --- | --- | --- | --- | --- | --- |
| **Southern** | Weaning | Post wean | 83 | 33 | 0.00** | 4.6(2.6-8.1) |
|  |  | Pre wean | 23 | 266 |  | Ref |
|  | Altitude | Plain | 54 | 263 | 0.00** | 8.8 (2.36-37.5) |
|  |  | Hilly | 2 | 86 |  | Ref |
|  | Water source | Deep & shallow well | 54 | 263 | 0.00** | 8.8 (2.36-37.5) |
|  |  | Spring | 2 | 86 |  | Ref |
|  | Presence of heater /cooler | No | 54 | 263 | 0.00** | 8.8 (2.36-37.5) |
|  |  | Yes | 2 | 86 |  | Ref |
|  | Disinfectant | Weekly | 17 | 59 | 0.01* | 2.6 (1.3-5.1) |
|  |  | Occasional | 13 | 57 |  | 2.0 (0.98.- 4.2) |
|  |  | Daily | 26 | 233 |  | Ref |
| **Northern** | Weaning | Post wean | 21 | 65 | 0.03** | 2.0 (1.1-3.7) |
|  |  | Pre wean | 31 | 193 |  | Ref |
|  | Presence of other animals in farm | Yes | 39 | 230 | 0.00** | 2.78(1.3-5.7) |
|  |  | No | 13 | 28 |  | Ref |
|  | Disinfectant | Weekly | 21 | 60 | 0.00** | 2.30 (1.5-3.52) |
|  |  | Occasional | 22 | 49 |  | 3.28 ( 2.0-5.38) |
|  |  | Daily | 9 | 149 |  | Ref |
|  | Ventilation | Fair | 22 | 49 | 0.00** | 3.13(1.67-5.9) |
|  |  | Good | 30 | 209 |  | Ref |
|  | Water source | Deep & shallow well | 13 | 28 | 0.00** | 4.8(2.4-9.7) |
|  |  | shallow well | 22 | 49 |  | 4.9(2.2-11.3) |
|  |  | Deep well | 17 | 181 |  | Ref |
|  | Presence of heater /cooler | No | 22 | 49 | 0.00** | 3.13 (1.66 -5.88) |
|  |  | Yes | 30 | 209 |  | Ref |
|  | Feed | Commercial | 35 | 77 | 0.00** | 3.6( 2.2 -6.2) |
|  |  | Own mill | 17 | 181 |  | Ref |
|  | Season | Winter | 1 | 9 | 0.00** | 0.89(0.1-7.4) |
|  |  | Summer | 29 | 72 |  | 3.2 (1.75-6.0) |
|  |  | Monsoon | 22 | 177 |  | Ref |
| **North East** | Weaning | Post wean | 13 | 16 | 0.00** | 9.5(3.8 -24) |
|  |  | Pre wean | 13 | 152 |  | Ref |

Ref- Reference category

* p≤0.05

**p≤ 0.01

**Supplementary table 4: Chi-square / Fisher’s exact test analysis of antibiotic resistance pattern of *E. coli* isolates (n=531) with various epidemiological factors**

| **Antibiotics** | **Susceptibility pattern** | **Sex** | | | **Health status** | | | **Weaning** | | |
| --- | --- | --- | --- | --- | --- | --- | --- | --- | --- | --- |
|  |  | **Male**  **(268)** | **Female**  **(263)** | ***X*^2^value** | **Diarrhoeic (93)** | **Non- Diarrhoeic (438)** | ***X*^2^value** | **Pre-wean (316)** | **Post wean (215)** | ***X*^2^value** |
| AMX | S | 22 | 20 | 0.07^NS^ | 12 | 30 | 3.86* | 15 | 27 | 10.72** |
|  | R | 246 | 243 |  | 81 | 408 |  | 301 | 188 |  |
| CTX | S | 49 | 46 | 0.06 ^NS^ | 11 | 84 | 2.82 ^NS^ | 35 | 60 | 24.67*** |
|  | R | 219 | 217 |  | 82 | 354 |  | 281 | 155 |  |
| CAZ | S | 49 | 55 | 0.58 ^NS^ | 12 | 92 | 3.12 ^NS^ | 39 | 65 | 26.00*** |
|  | R | 219 | 208 |  | 81 | 346 |  | 277 | 150 |  |
| COT | S | 178 | 184 | 0.77 ^NS^ | 45 | 317 | 20.34*** | 198 | 164 | 10.94*** |
|  | R | 90 | 79 |  | 48 | 121 |  | 118 | 51 |  |
| C | S | 167 | 174 | 0.85 ^NS^ | 43 | 298 | 15.87*** | 201 | 140 | 0.13^NS^ |
|  | R | 101 | 89 |  | 50 | 140 |  | 115 | 75 |  |
| GEN | S | 54 | 47 | 0.45 ^NS^ | 14 | 87 | 1.15 NS | 56 | 45 | 0.86 ^NS^ |
|  | R | 214 | 216 |  | 79 | 351 |  | 260 | 170 |  |
| TE | S | 182 | 174 | 0.18 ^NS^ | 67 | 289 | 1.28 ^NS^ | 216 | 140 | 0.61 ^NS^ |
|  | R | 86 | 89 |  | 26 | 149 |  | 100 | 75 |  |
| F/M | S | 182 | 188 | 0.80 ^NS^ | 72 | 298 | 6.93** | 245 | 125 | 22.77*** |
|  | R | 86 | 75 |  | 21 | 140 |  | 71 | 90 |  |
| NOR | S | 89 | 97 | 0.79 ^NS^ | 35 | 151 | 0.34 ^NS^ | 97 | 89 | 6.43* |
|  | R | 179 | 166 |  | 58 | 287 |  | 219 | 126 |  |
| ATM | S | 165 | 174 | 1.21 ^NS^ | 67 | 272 | 3.29 ^NS^ | 185 | 154 | 9.49** |
|  | R | 103 | 89 |  | 26 | 166 |  | 131 | 61 |  |
| CIP | S | 81 | 96 | 2.35 ^NS^ | 32 | 145 | 0.06 ^NS^ | 115 | 62 | 3.29 ^NS^ |
|  | R | 187 | 167 |  | 61 | 293 |  | 201 | 153 |  |
| FEP | S | 114 | 104 | 0.49 ^NS^ | 28 | 190 | 6.58* | 118 | 100 | 4.45* |
|  | R | 154 | 159 |  | 65 | 248 |  | 198 | 115 |  |
| CFM | S | 119 | 118 | 0.01 ^NS^ | 35 | 202 | 0.44 ^NS^ | 129 | 108 | 2.52  ^NS^ |
|  | R | 149 | 145 |  | 58 | 287 |  | 204 | 130 |  |
| CRO | S | 114 | 109 | 0.07 ^NS^ | 39 | 184 | 0.00^NS^ | 119 | 104 | 6.03** |
|  | R | 154 | 154 |  | 54 | 254 |  | 197 | 111 |  |
| CEF | S | 93 | 94 | 0.06 ^NS^ | 36 | 151 | 0.60 ^NS^ | 112 | 75 | 0.02 ^NS^ |
|  | R | 175 | 169 |  | 57 | 287 |  | 204 | 140 |  |

AMX- amoxicillin; CTX- cefotaxime, CAZ- ceftazidime, COT- cotrimazole , C- cholramphenicol, GEN- Gentamicin, TE- Tetracycline, F/M- nitrofurantoin, NOR- norfloxacin, ATM-aztreonam , CIP-Ciproflaxacin, FEP- cefepime, CFM- Cefixime, CRO-Ceftriaxone, CEF-Cefoperazone

NS-non significant; * - p≤0.05; ** - p≤ 0.01; *** - p ≤ 0.001

**Supplementary table 5: MAR indices of *E.coli* isolated from piglet fecal samples (n=531)**

| **S.no** | **MAR Index** | **Number of isolates (%)** |
| --- | --- | --- |
| 1 | 0.1 | 18 (3.39) |
| 2 | 0.2 | 73 (13.75) |
| 3 | 0.3 | 142 (26.74) |
| 4 | 0.4 | 62 (11.68) |
| 5 | 0.5 | 48 (9.04) |
| 6 | 0.6 | 75 (14.12) |
| 7 | 0.7 | 67 (12.62) |
| 8 | 0.8 | 11 (2.07) |
| 9 | 0.9 | 21 (3.95) |
| 10 | 1 | 14 (2.64) |

**Questionnaire for collection of data about pig farms**

**DIVISION OF EPIDEMIOLOGY**

**Indian Veterinary Research Institute (IVRI)**

**Izatnagar, Bareilly- 243 122 (UP)**

**Proforma for collection of information on organized swine farm**

**Date:…………………..**

**Details of farm**

Name of the farm :

Address :

Location :

Longitude :

Latitude :

Number of sheds :

Average number of breeding sows :

Average number of breeding boars :

Number of farm workers :

Litter size at birth (LSB) :

Litter size at weaning (LSW) :

Weaning age :

Feeding and Management :

1. concentrate
2. concentrate with fodder
3. swill feeding

Floor : Kachcha/Pakka/Concrete

Hygiene : Good/Normal/Poor

Colostrum feeding : Yes/No

Green feeding : Yes/No

Roughage feeding : Yes/No

Use of antibiotics in feed : Yes/No

Class of antibiotics used for treatment:

History of diarrhoea :

History of antibiotic resistance : Yes/No

Bedding used in the weaner pens : Yes/No

Type of bedding:

Feed : commercial/own mill

Water source : Shallow Well/deep well/spring

Temperature control in weaner pens : Yes/ No

How often are the weaner pens cleaned:

Use of disinfectants : Daily/Weekly/Occasional

**Individual piglet level data**

Breed : Desi/Cross bred/Pure bred

Sex : Male/ Female

Age (Days) :

Colostrum fed : Yes/No

Diarrhoea : Yes/ No

Frequency of diarrhoea :

1. 2 to 5 times / day b. More than 5 times /day

Type of fecal sample :

1. Watery b. Semi-solid c. Normal

Last date of antibiotic treatment :

Antibiotic used :

Deworming history : Yes/No

If yes, class of dewormer used

**Details of farm workers**

1. Average years of working of workers in the swine farm:
2. Less than one year b. 2 to 5 year c. more than 5 years
3. Age of the farm worker :
4. Between 20 to 30 years.
5. between 31 to 40 years
6. c. above 41 years
7. Health status of the workers : If any illness mention it
8. Any type of surgery performed :
9. Antibiotics used :
10. Separate handlers for adult and piglets : yes/no
11. Separate handlers for healthy and diseased piglets : yes/no

**(Signature of Veterinarian/data collector)**
